# Health system interventions to floods and heatwaves for maternal and child health services: a realist-informed systematic review protocol

**DOI:** 10.1136/bmjopen-2026-123914

**Published:** 2026-09-11

**Authors:** Sisay E. Debele, Fiammetta Maria Bozzani, Grace Mushinda-Musonda, Sari Kovats, Gabrielle Bonnet, Chitalu Miriam Chama-Chiliba, Anna M Foss, Everton Nunes da Silva, Josephine Borghi

**Affiliations:** 1 Department of Global Health and Development, Faculty of Public Health and Policy, London School of Hygiene & Tropical Medicine, London, UK; 2 Institute of Disaster Risk Management and Food Security Studies, Bahir Dar University, Bahir Dar, Amhara, Ethiopia; 3 Centre for Economic and Social Research, University of Zambia, Lusaka, Zambia; 4 Department of Public Health, Environments and Society, Faculty of Public Health and Policy, London School of Hygiene & Tropical Medicine, London, UK; 5 Clinical Research Department, Faculty of Infectious and Tropical Diseases, London School of Hygiene & Tropical Medicine, London, UK; 6 Faculty of Ceilândia, University of Brasília, Brasília, DF, Brazil; 7 International Institute for Applied Systems Analysis, Laxenburg, Austria

**Keywords:** Health policy, Health, Community child health, Health Services Accessibility, Climate Change

## Abstract

**Abstract:**

**Introduction:**

Floods and heatwaves are becoming more frequent and intense and can disrupt routine maternal and child health (MCH) services. Previous reviews have not systematically examined how context and mechanisms may shape adaptation outcomes. This realist-informed systematic review examines how, why and under what conditions interventions support access to, utilisation of and continuity of routine MCH services during flood and heat events.

**Methods and analysis:**

The search strategy was developed by integrating terms from 17 related reviews, refined with the author team and checked by an experienced London School of Hygiene & Tropical Medicine librarian. The initial database search was conducted on 16 May 2025 and the search was updated on 30 April 2026. Eight databases were searched: Web of Science, Ovid MEDLINE, EMBASE, Global Health, EconLit, GreenFILE, CINAHL and ProQuest Environmental Science & Public Policy. Records published between January 2000 and April 2026 were eligible. Search results were imported into EndNote and deduplicated. Title-and-abstract screening and full-text assessment were conducted independently by two reviewers for the initial search and are ongoing for the updated search, with ASReview being used to prioritise records during title-and-abstract screening. Data extraction across the full review evidence base remains ongoing using a structured template covering study context, intervention characteristics, mechanisms, outcomes, costs and implementation conditions. Study settings will be classified by country income group, health system context and Köppen–Geiger climate zone to compare evidence across settings and, where appropriate, to identify possible climate analogues for exploratory, context-specific assessment. Climate-zone similarity will not be used to infer intervention transferability or future effectiveness. The synthesis will be guided by the WHO Climate-Resilient Health Systems framework, Meadows’ leverage-points framework and realist-informed C–M–O reasoning. Interventions will be grouped to identify where they operate within the health system and which health system components and vulnerabilities they address. Findings will be reported in accordance with Preferred Reporting Items for Systematic Reviews and Meta-Analyses (PRISMA) guidance.

**Ethics and dissemination:**

Ethical approval is not required because the review uses published literature and does not involve human participants. The findings will be disseminated through a peer-reviewed systematic review publication. They will inform the design, improvement and scaling of interventions intended to maintain routine MCH services and strengthen health system resilience to floods and heatwaves across diverse settings.

**Registration details:**

The review was not prospectively registered with PROSPERO.

**STRENGTHS AND LIMITATIONS OF THIS STUDY**

- The review draws on global evidence, with no restrictions on geographic setting or language, and considers adaptation interventions implemented across multiple sectors.
- ASReview, an active-learning machine-learning tool, supports efficient prioritisation of the large evidence base during title-and-abstract screening, while all eligibility decisions are made independently by two reviewers.
- Interventions will be systematically classified and compared in relation to reported health-service delivery, utilisation, coverage and continuity-of-care outcomes using complementary health system and realist-informed analytical frameworks.
- Restricting eligibility to peer-reviewed studies may omit relevant interventions documented in government, World Health Organization, humanitarian and other organisational reports.
- Comparisons across climate and health system settings will be exploratory because similar climate zones and health system settings do not necessarily imply similar intervention effects, as implementation and other contextual factors may modify these effects.

## Background

 Climate change is already affecting health systems in many regions of the world. Flood risk is changing in many settings, and periods of extreme heat are becoming more frequent and intense.^1 2^ Climate projections suggest that global temperatures could rise by more than 1.5 °C above pre-industrial levels by the middle of the century and approach 2 °C if emissions continue on their current trajectory.^3 4^ Pregnant women, newborns and young children are among the populations most vulnerable to climate-related health risks. Extreme heat has been associated with adverse maternal and newborn outcomes, including gestational diabetes, pre-eclampsia, preterm birth, low birth weight, stillbirth and disruption to infant feeding practices.^5^ Routine maternal and child health (MCH) services, including antenatal, delivery, postnatal, neonatal, immunisation and routine child healthcare, are therefore important for protecting health during and after climate-related extreme weather events. This is particularly important for pregnant women and children because continuity of care often depends on regular and timely contact with health services, making routine care especially vulnerable to disruption during extreme events. Climate-related hazards can affect MCH systems through interacting demand- and supply-side pathways. On the demand side, recent epidemiological studies have reported associations between prenatal or early-life exposure to ambient temperature and other meteorological conditions and childhood allergic diseases, asthma and pneumonia.^6–8^ Although these studies did not examine health-service delivery or utilisation, their findings indicate that climate-related exposures may increase childhood morbidity and consequently the need for healthcare. On the supply side, floods can damage healthcare infrastructure, interrupt medical supply chains and cause power outages, while heat can disrupt cold chains and medicine storage, reduce workforce capacity and worsen patient care experiences. An increase in healthcare need may therefore occur at the same time that floods and heatwaves reduce the capacity of health systems to provide accessible and continuous care. In line with this, the World Health Organization (WHO) and United Nations climate summit declarations emphasise the importance of implementing adaptation strategies to reduce climate risks to health systems, while taking account of the needs of vulnerable populations such as pregnant women and children.^5 9 10^ To support countries in selecting effective and locally appropriate adaptation strategies, stronger evidence is needed on which interventions help protect routine MCH services during floods and heatwaves, whether they are effective and cost-beneficial and how they work in different contexts.

A recent realist-informed systematic review showed that floods and heatwaves disrupt MCH service use through six main pathways: restricted travel, displacement, income loss, increased health risks, disruption to health services and wider infrastructure disruption.^11^ The pattern differed by hazard: heat-related evidence was concentrated in emergency and inpatient care, whereas flood-related evidence more often pointed to disruption of routine MCH services such as antenatal, delivery and postnatal care as well as delays in emergency obstetric care access.^11^ There is also a wider body of work on adaptation to heat and flood risks. This includes facility and infrastructure measures such as cooling, ventilation, backup electricity and water systems, occupational health and workforce measures and service-delivery or governance responses.^12–15^ What is less clear is which interventions help maintain routine MCH services during floods and heatwaves and how their effects vary across health system and climatic settings.

Previous reviews have mapped a wide range of climate adaptation and health system resilience responses, including emergency preparedness, early warning and surveillance systems, climate-resilient infrastructure, facility adaptation, workforce training and protection, service-delivery changes, governance and planning measures and broader health system strengthening. However, these reviews do not answer the specific question addressed in this protocol. Some are limited by geographic focus, including reviews centred on Africa or Asia, while others focus on particular parts of the health system, such as hospitals, primary care, the health workforce, surgical systems or general health system resilience.^12 15–23^ Many of these reviews describe adaptation actions, implementation barriers or enabling conditions, but they do not consistently synthesise intervention effects on health service delivery, utilisation, coverage or continuity of care and they rarely focus specifically on routine MCH services during floods and heatwaves. A further limitation is that mechanisms and context are often underdeveloped. This matters because adaptation interventions are complex and may work differently depending on health system capacity, governance arrangements, workforce and infrastructure conditions, hazard characteristics, financing and local climate context. A realist-informed approach is therefore appropriate because it allows the review to move beyond whether interventions are reported to work and to examine how, why and under what conditions their effects on MCH service delivery and utilisation occur. This review therefore focuses on interventions implemented in the context of flood and/or heat risks for which there is evidence of impact on MCH services, with continuity of routine MCH service delivery and utilisation as the main outcomes of interest.

## Aim and objectives

The aim of this review is to synthesise evidence on interventions designed to reduce disruption to MCH service delivery and utilisation during floods and heatwaves and to examine the mechanisms through which they produce these effects in different contexts. The review will address the following specific objectives:

1. To systematically identify and classify interventions (strategies, measures and/or policies) for which there is evidence of impact on health service delivery and utilisation during heatwaves and flood events, including continuity of care for pregnant and postpartum women and children.
2. To assess the mechanisms through which interventions produce their effects and the role of contextual factors, including geography and broader health system characteristics, in shaping these effects, including enablers, barriers and unintended consequences.
3. To document, where such evidence is available, the costs or cost-effectiveness of interventions and their co-benefits or disbenefits, including climate mitigation, environmental, economic and health impacts.
4. To identify gaps in the evidence on adaptation interventions intended to protect routine MCH services during floods and heatwaves, including the intervention types, outcomes, costs, implementation conditions and health system and climate contexts that have been studied.

## Methods

### Study design

This manuscript reports the protocol and analytical plan for an ongoing systematic review informed by realist principles. The protocol was developed alongside the review activities. The initial database search was conducted on 16 May 2025 and covered evidence available up to that date. The search was updated on 30 April 2026 to identify additional records published or indexed between 17 May 2025 and 30 April 2026. Data extraction commenced in March 2026 and remains ongoing. At the time of the original submission to BMJ Open on 9 June 2026, title-and-abstract screening, full-text assessment and data extraction for records identified through the updated search were ongoing; these activities remain ongoing at the time of this revision. Data extraction across the full review evidence base, including studies identified through both the initial and updated searches, remains ongoing. Methodological appraisal, context-mechanism-outcome (C–M–O) informed synthesis, integration of the analytical frameworks, final synthesis and reporting are planned. No review findings, analytical results or conclusions are presented in this protocol. The review was not prospectively registered with PROSPERO because data extraction had commenced before registration was considered.

### Analytical framework

To guide this work, we draw on an analytical framework that combines three components: Meadows’ leverage points for systems transformation,^24^ the WHO climate-resilient health system building blocks^25^ and realist C-M-O reasoning.^26 27^ This review is realist-informed rather than a full-realist review. In this review, ‘realist-informed’ means that C–M–O reasoning informs data extraction and will guide interpretation and synthesis, while searching and study selection follow predefined systematic review procedures, and methodological appraisal will be conducted using the design-specific criteria of the Mixed Methods Appraisal Tool (MMAT). The realist elements adopted are C–M–O-informed data extraction, cross-study comparison and explanatory narrative synthesis.

**Figure 1:**
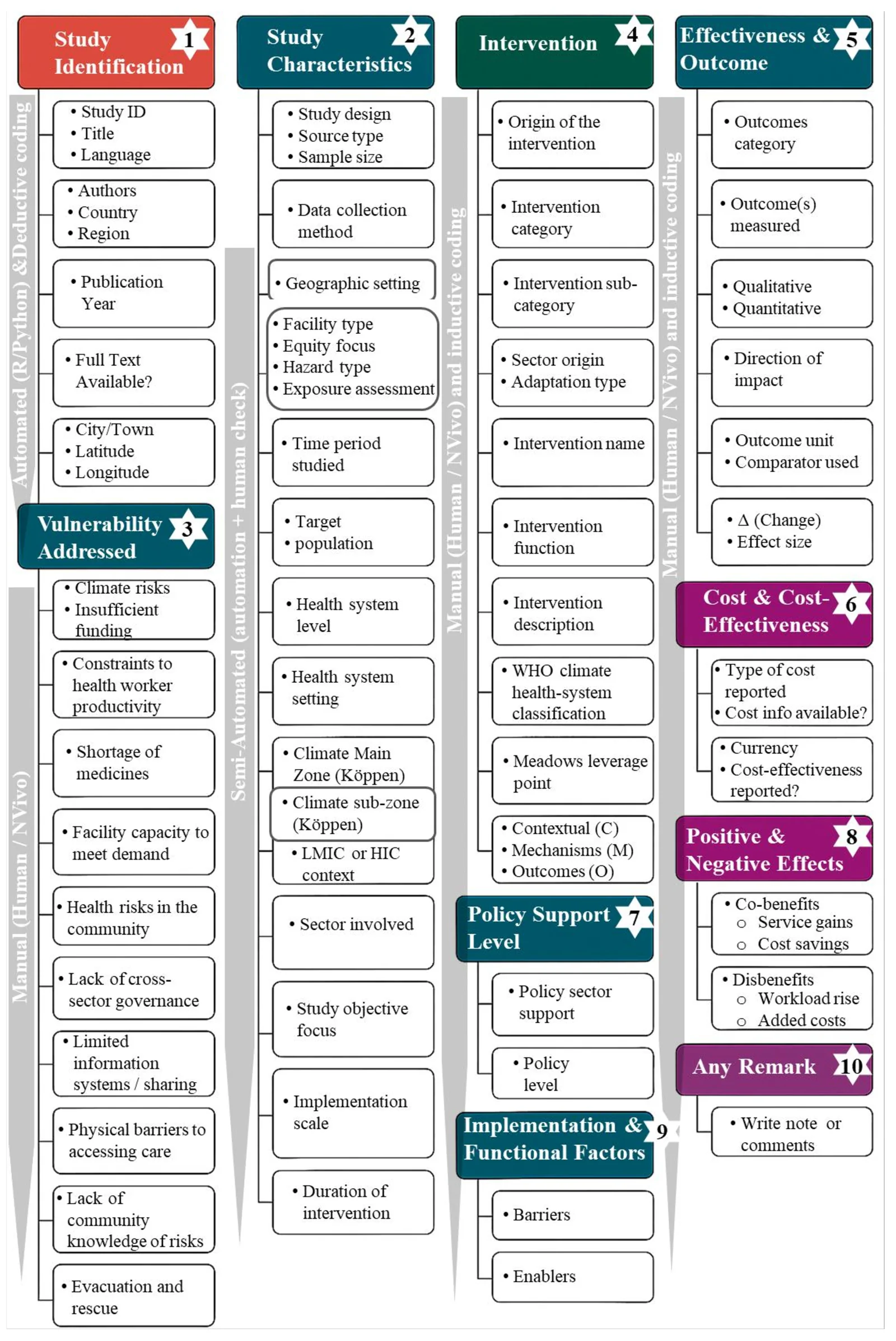
Data extraction framework used in this realist-informed systematic review. The figure summarises 10 domains included in the structured Excel extraction template and gives examples of the variables captured in each domain. Within study characteristics, hazard type and exposure assessment are recorded to capture how floods and heatwaves are defined and measured across studies. The figure also shows which fields are extracted automatically, semi-automatically or manually.

Together, these frameworks will be used to examine how interventions function in practice, the mechanisms they trigger and the parts of the health system they strengthen. Interventions will be grouped into thematic areas based on intervention type and the system vulnerabilities they address and then mapped against 10 components of the WHO framework and the 12 leverage points described by Meadows.^24^ The vulnerabilities considered in this review are those identified in a previous systematic review of flood and heat impacts on the delivery of and access to MCH services and are summarised in the ‘Vulnerability Addressed’ domain of figure 1.^11^

The structured extraction template records intervention characteristics and functions, contextual factors, reported or cautiously inferred mechanisms, outcomes, barriers, enablers, policy support, co-benefits and disbenefits in separate fields. Contextual factors include climatic and hazard characteristics, health system, economic, geographic, social and governance conditions. Climatic and hazard context will include Köppen–Geiger climate classification and, where reported, characteristics of the flood or heat event, such as hazard type, temperature or intensity, magnitude, duration and timing. In this review, a mechanism is understood as an underlying response or causal process through which an intervention may contribute to a health-service outcome in a particular context. Intervention inputs, design features, implementation activities and health system functions will not themselves be treated as mechanisms. For each reported health-service outcome, the synthesis will describe narratively the relevant context, the intervention and the changes it introduces and the mechanism reported by study authors or, where supported by the available evidence, cautiously inferred by the review team. Inferred mechanisms will be distinguished from author-reported mechanisms, and an association between an intervention and an outcome alone will not be considered sufficient evidence of a mechanism. These C–M–O-informed relationships will be compared within and across intervention categories and settings to identify recurring, contrasting and disconfirming explanatory patterns.

Study settings will also be classified by country income group; health system context, including degree of health-sector decentralisation and universal health coverage and Köppen–Geiger climate zone.^28 29^ These classifications will be used to describe and compare the climatic and health system contexts in which interventions have been reported. For interventions whose implementation or performance may be sensitive to climatic conditions, projected changes in Köppen–Geiger classification will be considered descriptively to identify possible climate analogues. A possible climate analogue will be defined as a setting whose projected future climate classification becomes similar to the present-day classification of a setting in which a comparable intervention has previously been reported with favourable health-service delivery or utilisation outcomes.^29–31^ The identification of a possible climate analogue will be treated as an exploratory, hypothesis-generating signal that an intervention may warrant further context-specific assessment. It will not be interpreted as evidence that the intervention is transferable or will produce comparable outcomes in the future setting. Climatic similarity will be considered alongside country income, health system organisation and capacity, governance, infrastructure, workforce availability, service access, geographic and social conditions, intervention design and implementation requirements. Climate-zone similarity will therefore be treated as one dimension of context rather than as a sufficient basis for recommending the transfer of an intervention.

The WHO climate-resilient health system framework and Meadows’ leverage-points framework provide complementary analytical structures. The WHO framework will be used to identify where interventions operate within the health system and the vulnerabilities they address, while Meadows’ framework will be used to characterise the depth of system change represented by an intervention, ranging from relatively limited adjustments to system inputs and parameters to broader changes in system goals, structures or power relations.^24 25^ These frameworks and classifications support the C–M–O-informed synthesis but will not themselves be treated as mechanisms or as components of realist methodology.

Realist principles will guide the synthesis of evidence on why particular interventions may contribute to outcomes in some contexts but not others and on the health system mechanisms through which those outcomes may arise.^22 32 33^ The review does not develop or test formal C–M–O configurations or an overarching programme theory and does not use iterative theory-driven searching, purposive theoretical sampling or realist relevance-and-rigour appraisal. These elements were not adopted because the review was designed to systematically identify and classify empirical evidence across multiple intervention categories and to examine mechanisms and contextual influences across a heterogeneous evidence base, rather than to develop and refine a single predefined programme theory. A predefined systematic search was therefore used, and methodological quality will be assessed using the MMAT to provide a consistent, design-specific appraisal of qualitative, quantitative and mixed-methods studies.^34^ Realist reasoning will guide interpretation and synthesis but will not replace the systematic procedures used to identify, select and appraise the evidence.

### Search terms and strategy

The initial database searches were conducted on 16 May 2025, and the search was updated on 30 April 2026. The search strategy combines indexing terms (eg, MeSH) and free-text keywords covering three core concepts: (a) floods and heatwaves; (b) interventions including flood and heat preparedness and response measures and (c) health system outcomes, including service delivery, utilisation and access to care. Development of the search terms drew on 17 systematic and scoping reviews on adaptation and health systems (see online supplemental table S1), including a recent scoping review on health system adaptation to floods and extreme heat.^35^ Key terms related to floods, heatwaves and adaptation were reviewed to guide development of the final search terminology. The full database-specific search strategies are provided in online supplemental table S2. Initial search results were screened to confirm that key studies identified in previous reviews were retrieved. Where these were not captured, additional keywords and synonyms were incorporated into the search strategy to improve coverage of climate hazards, adaptation measures and health system outcomes. The search was limited to records published between January 2000 and April 2026.

The search was conducted across eight bibliographic databases: Web of Science, Ovid MEDLINE, EMBASE, Global Health, EconLit, GreenFILE, CINAHL and ProQuest Environmental Science & Public Policy. Together these databases capture a broad range of literature spanning biomedical research, health systems, environmental hazards, disaster risk and climate adaptation policy. With assistance from a research librarian, the search strategy was adapted for each database to reflect its indexing system and controlled vocabulary. All retrieved records were exported in RIS format and imported into EndNote 21 for initial deduplication using the built-in Find Duplicates function. Of the 38 378 records retrieved, 4270 duplicates were removed at this stage, leaving 34 108 records. R was used as a second deduplication step because EndNote’s built-in duplicate detection relies on selected bibliographic fields and may miss duplicate records where titles, author names, punctuation or DOI fields differ slightly between databases. The remaining records were exported from EndNote in RIS format and processed in R V.4.3.1 using the bibliometrix and stringdist packages. DOI values were standardised and checked for exact matches where available. Titles were converted to lowercase, and punctuation and additional spaces were removed. Candidate record pairs were then compared using the Jaro–Winkler distance metric.^36 37^ Pairs with a distance of 0.05 or less, corresponding to at least 95% title similarity, were flagged as potential duplicates and manually checked against publication year, authors and DOI before removal. This second stage identified 1237 additional duplicates. Following manual verification, 32 871 unique records remained. Files containing the identified duplicates and the final deduplicated dataset were retained in RIS and CSV formats to provide an audit trail of the process.

### Eligibility criteria

Studies are eligible if they report interventions, strategies, policies or measures designed to reduce the impact of floods or heatwaves on health service delivery, access or utilisation, with relevance to MCH. For this review, MCH services refer to services for pregnant and postpartum women, newborns and children under five, with a particular focus on continuity of routine care. This includes services such as antenatal, delivery, postnatal, neonatal, immunisation and other routine child health services. Eligible studies may use qualitative, quantitative or mixed-methods designs and must report empirical evidence on the effects of adaptation interventions on the specified outcomes. Studies focusing exclusively on health outcomes without reference to health system functioning or service delivery are excluded. Literature reviews, meta-analyses, editorials, commentaries, opinion pieces, books or book chapters and conference abstracts are also excluded. Full inclusion and exclusion criteria are presented in table 1.

**Table 1:** Inclusion and exclusion criteria

Inclusion criteria	Exclusion criteria
Hazard focus – The study must address floods and/or high-temperature or heatwave-related hazards or broader adaptation interventions designed to strengthen resilience and adaptive capacity with the potential to mitigate flood or heat risks.	Studies not addressing floods and/or heat.
Adaptation interventions – The intervention can come from within or outside the health sector but must have measured impact on health service delivery and/or utilisation.	Studies focused solely on climate mitigation, financial reporting or theoretical frameworks without applied adaptation strategies.
Study design – The study should be original research that presents empirical evidence (quantitative, qualitative or mixed methods) or a model based on empirical evidence.	Literature reviews, systematic reviews, meta-analyses, opinion pieces, editorials, commentaries, books or book chapters and conference abstracts.
Target populations – The study must assess interventions or strategies that provide direct or indirect benefits to the delivery of or access to services for pregnant women and/or children under five. Interventions that reduce disruption to overall health service delivery are also eligible, even if mothers and children are not explicitly targeted, provided these interventions may reasonably benefit mothers or children.	Studies of populations with no direct or indirect benefit to services for pregnant women or children under five (eg, interventions targeting only men or only elderly people).
Measurable outcomes – The study must report at least one health system outcome (quantitative or qualitative), including: healthcare service delivery, service utilisation, service coverage or indicators reflecting the ability of health services to maintain or restore service provision during climate-related disruptions.	Studies lacking measurable outcomes related to health service delivery, utilisation, coverage or resilience.
Publication timeframe and accessibility – The full text must be available. Only studies published between January 2000 and April 2026 are eligible. This period was selected to capture contemporary evidence on climate adaptation, health system resilience and service-delivery responses developed during the period in which climate change adaptation became more prominent in global health and health system policy. There will be no restrictions on geographic scope or language.	Studies published before January 2000, where the full text is not accessible or where data are insufficient for evaluation.
Unique records – Studies included after deduplication.	Duplicate publications or studies presenting redundant or overlapping data without new or additional insights.

### Selecting and appraising documents

After deduplication in EndNote and R, all references were imported into ASReview Lab, an open-source tool that uses active learning to prioritise records likely to be relevant.^38^ Screening was guided by the inclusion and exclusion criteria (table 1). For records identified through the initial search, titles and abstracts were screened independently by two reviewers, with prioritisation supported by ASReview, which ranked references according to predicted relevance to support efficient identification of potentially eligible studies. A small training set of 10 eligible and 10 ineligible studies was used to initialise the model. Records were divided into seven assigned screening sets, each assessed independently by two reviewers. One reviewer participated across all seven sets to support consistent application of the eligibility criteria, while the second reviewer differed between assigned sets. Records were screened iteratively in the order prioritised by ASReview. Within each set, screening continued until reviewers encountered an uninterrupted sequence of irrelevant records equivalent to 10% of the records assigned to that set. Across the seven screening sets, this corresponded to 355–822 consecutive irrelevant records, depending on set size. This set-size-proportional threshold was used as a pragmatic operational stopping heuristic informed by approaches to active-learning-assisted screening rather than as a universally validated cut-off.^39^ ASReview prioritised the presentation of records, but all eligibility judgements were made by the reviewers. Title-and-abstract screening of records identified through the updated search is ongoing using the same independent dual-review and ASReview-assisted procedures. For the initial search, records considered potentially eligible were then retrieved for full-text assessment. Full texts were reviewed independently by two reviewers, and any disagreements were resolved through discussion. To maintain consistency across reviewer sets, one reviewer also reviewed all full-text records. Full-text assessment of potentially eligible records identified through the updated search is ongoing using the same independent dual-review procedures. All search files, deduplication logs and ASReview project files will be archived in a secure institutional repository and made available on request.

### Data extraction and synthesis

The review methods comprise four stages: extraction of study data, organisation and classification of extracted variables, assessment of study quality and synthesis of findings.

#### Data extraction

Data are being extracted into a structured Excel template organised around the 10 domains shown in figure 1: study identification, study characteristics, health system vulnerability addressed, intervention characteristics, effectiveness and outcomes, cost and cost-effectiveness, policy support level, positive and negative effects, implementation and functional factors and additional remarks. The template captures bibliographic details (eg, study ID, title, authors, publication year and language), study characteristics (eg, study design, data collection method, study setting, facility type, target population, climate hazard type and exposure assessment and study period) and contextual information, including geographic location, climate zone (Köppen classification), scale (eg, facility level), health system context and country income classification (LMIC or HIC). Where reported or clearly identifiable from the study description, the template also records the health system vulnerability that the intervention is intended to address, such as infrastructure constraints, health workforce limitations, supply-chain disruptions or barriers to service access.

Detailed information on intervention characteristics is being extracted, including the origin of the intervention, broad intervention category and more specific subcategory, sector of origin (eg, health, transport, agriculture), adaptation type, intervention description and intervention function. Intervention category refers to the broad type of action, such as emergency preparedness, service-delivery adaptation, workforce support, facility or infrastructure resilience, supply-chain strengthening, community outreach, social protection, digital technology, governance, financing or nature-based approaches. Intervention subcategory captures the more specific action described in the study. Adaptation type is being classified as absorptive, adaptive, transformative, mixed or not specified. Intervention function describes what the intervention is intended to do in relation to service continuity or health system vulnerability, such as maintaining access to care, protecting facility operations, supporting the workforce, preserving medical supplies or cold chains, improving information flows, managing demand, restoring services after disruption or reducing community-level risks affecting MCH care.

The extraction template further captures information on intervention outcomes, including the type of outcome measured, whether outcomes are reported qualitatively or quantitatively, the direction of impact and effect sizes where available. The extraction framework records study design, comparator use, direction of impact, reported change and any reported effect size separately. Comparator use is recorded as a control-group comparison, a pre–post comparison, no comparator or not reported. Direction of impact is recorded as positive, neutral, negative, mixed or not reported, while the change field records a reported delta, change from baseline, before–after difference, no change or not reported. These fields will be interpreted together with the underlying study design. The absence of a separate contemporaneous control group will not by itself determine the strength of evidence, as some quasi-experimental approaches, including interrupted time-series designs, use repeated observations and the pre-intervention outcome trajectory to provide a counterfactual comparison. Additional domains record cost and cost-effectiveness information, the level of policy support, reported co-benefits and/or disbenefits to health or emissions and barriers and enabling conditions. To support the realist-informed interpretation of the evidence, the extraction template also records C–M–O elements where these are reported or can be clearly identified in the studies. These fields will allow examination of how contextual conditions influence intervention mechanisms and outcomes across different health system, economic and climate settings. Structured bibliographic and geographic variables are being extracted automatically where possible using R or Python, as these elements are typically formatted in ways that lend themselves to automated extraction.^40^ Information on remaining domains is being extracted manually using the structured template to ensure consistent interpretation across studies.^41^

#### Organisation and classification of extracted variables

After extraction, studies will be organised according to the predefined variables included in the extraction template. Interventions will be grouped by intervention type, health system component and geographic setting. Using the information recorded in the extraction template, interventions will then be classified according to the WHO climate-resilient health system building blocks, allowing examination of how different adaptation strategies operate within specific parts of the health system. Interventions will also be mapped to Meadows’ leverage points. To assess the consistency of data extraction, a second reviewer will independently re-extract data from 20% of the included studies, selected to include both flood- and heat-related studies and the main study-design categories. Discrepancies will be discussed and resolved before synthesis, and the extraction guidance will be revised where necessary. If recurring or systematic discrepancies are identified, independent verification will be extended to additional studies and relevant data fields.

#### Quality and risk-of-bias assessment

The methodological quality of included studies will be assessed using the MMAT, which is suitable for reviews that include qualitative, quantitative and mixed-methods studies and allows appraisal across these designs using a single framework.^34^ Each study will be assessed using the MMAT criteria corresponding to its design category: qualitative studies, randomised controlled trials, non-randomised studies (including observational studies with statistical adjustment for confounding, quasi-experimental designs and interrupted time-series designs, where applicable), quantitative descriptive studies or mixed-methods studies. Each study will be assessed using the MMAT criteria relevant to its design. Quantitative descriptive studies will therefore be appraised separately from randomised, non-randomised, qualitative and mixed-methods studies. The strength of any inference about intervention effects will be qualified according to study design, the comparative or counterfactual structure of the evaluation and the relevant MMAT assessment. Quantitative descriptive studies will only be included where they meet the eligibility criteria and report empirical evidence relevant to the review outcomes. This assessment will help identify potential sources of bias and clarify the strengths and limitations of the available evidence.^42^

#### Data analysis and synthesis

The synthesis will first provide a descriptive overview of the literature, including the types of interventions identified, their geographic distribution, study characteristics and the health system and climate contexts in which they were implemented. For each vulnerability domain, the evidence on relevant interventions will be summarised, including their reported impacts on health service delivery, service utilisation and related health system outcomes. Evidence will be interpreted according to study design, the comparative or counterfactual structure of the evaluation and methodological quality. Randomised controlled trials and appropriately designed quasi-experimental evaluations, including natural experiments, interrupted time-series, controlled interrupted time-series, controlled before–after studies and other appropriate longitudinal comparative designs, will provide stronger evidence when assessing intervention effects than non-comparative descriptive studies. Quantitative or qualitative descriptive studies and other non-comparative studies may contribute evidence on intervention implementation, context, feasibility, barriers, enablers, reported service utilisation patterns, and reported mechanisms or evidence relevant to cautiously inferred mechanisms.

For the realist-informed component of the synthesis, mechanism-related information will be analysed separately from intervention characteristics and functions, barriers and enablers, and outcomes. Where a mechanism is not stated directly, cautious inference may draw on qualitative accounts, implementation findings, temporal relationships, differences between settings or population groups, barriers and enabling conditions, and explanations provided by study authors for why an intervention succeeded, failed or produced different outcomes. Interpretation will take account of study design and methodological quality. The synthesis will examine evidence across three dimensions: (1) the health-system vulnerabilities addressed, (2) the health-system domains in which interventions operate, and (3) the climatic, hazard and health-system contexts in which interventions have been implemented. Visualisation will include matrices linking health-system components with intervention types, diagrams illustrating C–M–O pathways, and graphical summaries of patterns across intervention categories, geographic regions and health-system vulnerabilities. R will be used for data management, analysis and visualisation, while Python will support selected automation and data-processing tasks.

## Discussion

This review addresses a heterogeneous evidence base spanning different interventions, study designs, hazard characteristics and settings. The principal methodological strengths and limitations of the review are discussed below. A key methodological strength of this realist-informed systematic review is its structured approach to characterising adaptation interventions using several linked analytical dimensions, supporting the review’s aim of identifying not only which interventions have been evaluated but also how and why they may work across different contexts. Interventions will be characterised according to the health system vulnerabilities they address, their type, function and sector of origin. They will then be mapped to the WHO climate-resilient health system framework to identify where they operate within the health system and to Meadows’ leverage points framework to characterise the depth of system change they represent.^24 25^ Climatic, hazard and health system context will be characterised using Köppen–Geiger climate classification alongside, where reported, characteristics of the flood or heat event, such as hazard type, temperature or intensity, magnitude, duration and timing, as well as country income, health system organisation and other contextual conditions.^28 29^ These dimensions will inform the C–M–O-informed narrative synthesis, allowing the review to examine how contextual conditions may shape the processes through which interventions contribute to reported MCH service delivery, access, utilisation, coverage and continuity outcomes.^26 27^ The broad geographic scope of the review and inclusion of interventions originating within and outside the health sector further enable comparison of adaptation approaches across diverse settings.

The review also incorporates several methodological features intended to support rigour and consistency. The search covered eight databases spanning biomedical research, health systems, economics, environmental hazards and climate adaptation, with no restrictions on geographic setting or language. Screening of records identified through the initial search was conducted independently by two reviewers, with ASReview used to prioritise records during title-and-abstract screening while eligibility decisions remained with the reviewers and with one reviewer participating across all screening sets to support consistent application of the eligibility criteria.^38 39^ The same independent dual-review and ASReview-assisted procedures are being applied to records identified through the updated search. Data are being extracted using a structured framework that supports consistent and comparable recording across studies of otherwise heterogeneous design, covering study characteristics, health system vulnerabilities, intervention characteristics, climatic and hazard contexts, health system contexts, mechanisms, outcomes, costs and implementation conditions. To assess the consistency of data extraction, a second reviewer will independently re-extract data from 20% of included studies, with discrepancies discussed and resolved before synthesis. Inclusion of qualitative, quantitative and mixed-methods evidence allows different dimensions of complex health system interventions to be examined, while methodological quality will be assessed using MMAT criteria appropriate to each study design.^34^

Nevertheless, the review has a few limitations. Restricting eligibility to peer-reviewed studies may omit adaptation interventions documented only in government, WHO, humanitarian and other organisational reports and may increase the potential influence of publication bias.^43^ In addition, the pragmatic stopping threshold used during ASReview-assisted title-and-abstract screening may have left some eligible lower-ranked records unscreened, because active learning prioritises records according to predicted relevance rather than guaranteeing that all potentially eligible records are encountered before screening stops.^38 39^ Differences in intervention types, study designs, outcome measures and settings may also limit direct comparisons between studies. Rigorous outcome evaluation may be challenging for interventions implemented during floods, heatwaves and other extreme events, where randomisation or conventional concurrent control groups may not be feasible. Appropriately designed quasi-experimental approaches, including natural experiments and interrupted time-series designs or observational studies will be drawn upon where available. Quantitative or qualitative descriptive and other non-comparative studies will also serve as sources of information on implementation, mechanisms, context and reported service utilisation changes, recognising the limitations of such evidence for causal attribution. Findings will therefore be interpreted according to study design, comparative or counterfactual structure and methodological quality rather than treating all forms of evidence as equivalent.

The Köppen–Geiger classification provides a standardised way of describing climatic context but cannot by itself establish whether an intervention is transferable between settings.^28 29^ Comparisons across climate and health system settings will therefore be exploratory, as even when climate zones and health system settings are comparable, this does not necessarily mean that intervention effects will be the same, as implementation and other contextual factors can moderate intervention effects. Locations with similar climatic classifications may differ substantially in hazard characteristics, health system organisation and capacity, governance, financing, infrastructure, workforce availability, service access, population needs and implementation conditions. The climate-analogue assessment will therefore be hypothesis-generating, identifying interventions that may warrant further context-specific assessment rather than establishing transferability or predicting future effectiveness.^29–31^ C–M–O-informed relationships and explanatory patterns will likewise be compared across, rather than generalised across, intervention categories and settings and interpreted as context-dependent explanations of how interventions may work under particular conditions rather than as evidence that the same intervention will necessarily produce the same outcomes elsewhere.^26 27^ These limitations mean that the review findings should be interpreted as identifying interventions and C–M–O-informed explanatory patterns that warrant further context-specific assessment rather than as definitive evidence that an intervention will be effective in another setting.

## Supplementary material

10.1136/bmjopen-2026-123914 online supplemental file 1

10.1136/bmjopen-2026-123914 online supplemental file 2
