# Supplementary material for: Health system interventions to floods and heatwaves for maternal and child health services: a realist-informed systematic review protocol
Source: BMJ Open. 2026 Sep 11;16(9):e123914. doi: 10.1136/bmjopen-2026-123914 (PMC13583685; doi:10.1136/bmjopen-2026-123914)
Supplement: Supplementary file 1 [file bmjopen-2026-123914.reviewer_comments.pdf]

PEER REVIEW HISTORY

BMJ Open publishes all reviews undertaken for accepted manuscripts. Reviewers are asked to complete a checklist review form and are provided with free text boxes to elaborate on their assessment. These free text comments are reproduced below.

ARTICLE DETAILS

Title (Provisional)

Health-system interventions to floods and heatwaves for maternal and child health services: a realist-informed systematic review protocol

Authors

Debele, Sisay Eshetu; Bozzani, Fiammetta Maria; Mushinda-Musonda, Grace; Kovats, Sari; Bonnet, Gabrielle; Chama-Chiliba, Chitalu Miriam; Foss, Anna M; da Silva, EN; Borghi, Josephine

VERSION 1 - REVIEW

**Reviewer**	**1**
**Name**	**Story, Lisa**
**Affiliation**	**King's College London**
**Date**	**13-Jul-2026**
**COI**	**None**

Thank you for asking me to review this manuscript.

I think this is an exceedingly important topic with clear implications globally.

I think the methodology is appropriate and outputs will have clear policy relevance for maternal and child health.

The protocol encompasses multiple databases, however it appears if the search has already been completed ‘At the time of writing, the search, deduplication, title and abstract screening, full-text assessment, and data extraction had been completed’. Which makes me question if this really is a protocol.

At a minimum details of the time of search, data extraction etc should be clearly given and why the search has already been done prior to publication of the protocol.

It is also unclear why it hasn’t actually been registered with PROSPERO yet as screening and data extraction seem to have been completed already.

The protocol does focus on peer reviewed publications but this may have limitations as some publications eg from governtments, WHO reports etc may document climate interventions

which may also be very relevant to the topic. Perhaps this could be added to the limitations section.

Why was 10% consecutive irrelevant records selected as a threshold. There is no justification for this number. Likewise why only 10% of extracted records verified and not dual extraction throughout or at least 20%?

The article sells itself as a realist informed review but explicitly stating which elements have been adopted and which haven't and why would be helpful for the reader.

Climate zone transferability claims are undoubtably very exciting but perhaps could be toned down somewhat as there are inherent differences between different regions not only related to the climate so perhaps the exploratory nature could be emphasised more?

**Reviewer**	**2**
**Name**	**Lu, Chan**
**Affiliation**	**Central South University, XiangYa School of Public Health**
**Date**	**13-Jul-2026**
**COI**	**None**

This manuscript presents a well-conceived protocol for a realist-informed systematic review on health-system interventions designed to protect routine maternal and child health (MCH) services during floods and heatwaves. The topic is highly timely and policy-relevant, given the accelerating impacts of climate change on health systems globally. The authors have developed a sophisticated analytical framework that integrates the WHO climate-resilient health systems building blocks, Meadows' leverage points, and realist C-M-O reasoning—a novel and theoretically grounded approach. The search strategy is comprehensive, covering eight databases with a well-documented search string. The use of ASReview for active-learning-assisted screening and dual-independent screening adds methodological rigor. However, there are some problems with this manuscript.

Due to the great meaning and a possibility in an improvement after a major revision, I suggest the editor accept this manuscript with major revision.

I have some comments and suggestions for this manuscript as follows:

1. The authors state that screening will stop once reviewers encounter a consecutive run of irrelevant records equivalent to 10% of records assigned to that set (page 13, lines 247–249). This is a pragmatic heuristic, but it is not a standardly validated stopping criterion in active-learning-assisted screening. Empirical studies on ASReview have shown that stopping rules vary widely, and a fixed percentage may either miss eligible records or lead to premature termination, depending on the prevalence of relevant records in the set.

2. The protocol states that C-M-O configurations will be developed, but the methods for deriving mechanisms from the included studies are not fully described. Realist reviews typically involve retroduction—inferring underlying mechanisms from patterns observed across studies. The protocol currently describes data extraction and classification but does not explain how the team will move from extracted data to C-M-O configurations.

3. The protocol includes quantitative descriptive studies (cross-sectional surveys, case series) alongside qualitative, RCT, and quasi-experimental studies. While this inclusivity is appropriate for a realist review, quantitative descriptive studies without comparison groups may provide limited evidence on intervention effects and may bias the synthesis if not critically appraised separately.

4. The protocol states that "at the time of writing, the search, deduplication, title and abstract screening, full-text assessment, and data extraction had been completed" (page 8, lines 158–160). This is unusual for a protocol submission and raises questions about whether the review is truly prospective. While retrospective protocols are not necessarily disallowed, transparency about what has already been done is critical.

5. Some recent study found significant associations of increased temperature or extreme heat with maternal and childhood chronic outcomes (e.g., allergic diseases, metabolic diseases) and infectious diseases (pneumonia, otitis media, COVID-19) (https://doi.org/10.1016/j.envint.2022.107456; https://doi.org/10.1016/j.buildenv.2024.112317; https://doi.org/10.1016/j.buildenv.2023.110668; https://doi.org/10.1016/j.buildenv.2023.110800). Furthermore, some other new studies explain the potential mechanisms underlying the associations, such as the expressions of transient receptor potential (TRP) ion channels (TRPV1, TRPV4, TRPA1, TRPM2) (https://doi.org/10.1016/j.jhazmat.2026.142315; https://doi.org/10.1016/j.envpol.2025.126321; https://doi.org/10.1111/all.15883). I suggest the authors to discuss your findings with (some of) the new articles and to further support your findings and discuss the potential mechanism to support your results.

VERSION 1 - AUTHOR RESPONSE

Reviewer #1

1.1 Thank you for asking me to review this manuscript.

Response:

We thank Reviewer #1 for taking the time to review the manuscript and for providing constructive comments.

1.2 I think this is an exceedingly important topic with clear implications globally.

Response:

We are grateful to Reviewer #1 for recognising the importance of this topic and its relevance across diverse global settings.

1.3 I think the methodology is appropriate and outputs will have clear policy relevance for maternal and child health.

Response:

We thank Reviewer #1 for this positive assessment of the methodological approach and for recognising the potential policy relevance of the review for maternal and child health services.

1.4 The protocol encompasses multiple databases, however it appears if the search has already been completed 'At the time of writing, the search, deduplication, title and abstract screening, full-text assessment, and data extraction had been completed'. Which makes me question if this really is a protocol.

Response:

We thank Reviewer #1 for raising this concern. We agree that the wording in the submitted manuscript was inaccurate because it suggested that all stages of the review had already been completed. This was not the case. The initial database search covered evidence available up to 16 May 2025. The search was subsequently updated on 30 April 2026 to identify additional records published or indexed between 17 May 2025 and 30 April 2026. At the time of the original submission to BMJ Open on 9 June 2026, title-and-abstract screening, full-text assessment and data extraction for records identified through the updated search were ongoing; these activities remain ongoing at the time of this revision. Data extraction across the full review evidence base, including studies identified through both the initial and updated searches, also remains ongoing. Methodological appraisal, C–M–O-informed synthesis, integration of the analytical frameworks, final synthesis and reporting are planned. We have therefore removed the inaccurate statement from the manuscript and revised the Study design section to distinguish clearly between the completed database searches and initial-search screening procedures and the updated-search screening, full-text assessment and review-wide data extraction activities that remain ongoing. The revised manuscript presents the protocol and analytical plan for an ongoing review and does not report review findings, analytical results or conclusions. The fuller review chronology is provided in our response to Reviewer #1, Comment #1.5, and the registration issue is addressed separately under Reviewer #1, Comment #1.6.

1.5 At a minimum detail of the time of search, data extraction etc. should be clearly given and why the search has already been done prior to publication of the protocol.

Response:

We thank Reviewer #1 for requesting a clearer account of the review timeline and an explanation of why review activities began before publication of the protocol. The protocol manuscript was developed alongside the review activities. It was initially submitted to BMJ Global Health on 1 May 2026 and was subsequently transferred to BMJ Open after we were advised on 5 June 2026 that BMJ Global Health did not publish research protocols. Review activities therefore continued while the protocol was undergoing submission and transfer. The initial database search was conducted on 16 May 2025 and covered evidence available up to that date. To ensure that more recent evidence was also captured, the search was updated on 30 April 2026 to identify additional records published or indexed between 17 May 2025 and 30 April 2026. Data extraction commenced in March 2026 and remains ongoing. At the time of the original submission to BMJ Open on 9 June 2026, title-and-abstract screening, full-text assessment and data extraction for records identified through the updated search were ongoing; these activities remain ongoing at the time of this revision. Data extraction across the full review evidence base, including studies identified through both the initial and updated

searches, also remains ongoing. Methodological appraisal, C–M–O-informed synthesis, integration of the analytical frameworks, final synthesis and reporting are planned. We have revised the Methods – Study design section to clarify the timing of the searches, the commencement of data extraction and the current status of the review while avoiding unnecessary operational detail. The revised text appears on page 7, lines 153–166 of the track-changed manuscript and reads as follows: "*This manuscript reports the protocol and analytical plan for an ongoing systematic review informed by realist principles. The protocol was developed alongside the review activities. The initial database search was conducted on 16 May 2025 and covered evidence available up to that date. The search was updated on 30 April 2026 to identify additional records published or indexed between 17 May 2025 and 30 April 2026. Data extraction commenced in March 2026 and remains ongoing. At the time of the original submission to BMJ Open on 9 June 2026, title-and-abstract screening, full-text assessment and data extraction for records identified through the updated search were ongoing; these activities remain ongoing at the time of this revision. Data extraction across the full review evidence base, including studies identified through both the initial and updated searches, remains ongoing. Methodological appraisal, C–M–O-informed synthesis, integration of the analytical frameworks, final synthesis and reporting are planned. No review findings, analytical results or conclusions are presented in this protocol. The review was not prospectively registered with PROSPERO because data extraction had commenced before registration was considered.*"

1.6 It is also unclear why it hasn't actually been registered with PROSPERO yet as screening and data extraction seem to have been completed already.

Response:

We thank Reviewer #1 for raising this point. As clarified in our response to Reviewer #1, Comment 1.5, the review had not been completed. Title-and-abstract screening and full-text assessment of records identified through the updated search remain ongoing. Data extraction across the full review evidence base, including studies identified through both the initial and updated searches, remains ongoing. However, data extraction had already commenced when prospective registration was considered, and the review was therefore not prospectively registered with PROSPERO. We have clarified the registration status in the Abstract and Methods. This issue is also addressed in our response to Editor Comment #2. The Abstract – Registration details section now states on page 2, line 48: *"Registration details The review was not prospectively registered with PROSPERO."* The Methods section now states on page 7, lines 165–166: *"The review was not prospectively registered with PROSPERO because data extraction had commenced before registration was considered."*

1.7 The protocol does focus on peer reviewed publications but this may have limitations as some publications eg from governtments, WHO reports etc may document climate interventions which may also be very relevant to the topic. Perhaps this could be added to the limitations section.

Response:

We thank Reviewer #1 for highlighting this limitation. We agree that restricting eligibility to peer-reviewed studies may omit relevant adaptation interventions documented in government, World Health Organization, humanitarian and other organisational reports. Grey literature was outside the scope of the review, and interventions reported only in these sources may therefore not be captured. We have added this limitation to both the Strengths and limitations of this study section and the Discussion. The Strengths and limitations of this study section now states on page 3, lines 61–63: "*Restricting eligibility to peer-reviewed studies may omit relevant interventions documented in government, World Health Organization, humanitarian and other*

organisational reports." The Discussion now states on page 22, lines 496–499: *"Restricting eligibility to peer-reviewed studies may omit adaptation interventions documented only in government, World Health Organization, humanitarian and other organisational reports and may increase the potential influence of publication bias [43]."*

1.8 Why was 10% consecutive irrelevant records selected as a threshold. There is no justification for this number. Likewise, why only 10% of extracted records verified and not dual extraction throughout or at least 20%?

Response:

We thank Reviewer #1 for raising these two points. The two references to 10% concerned separate procedures: the stopping threshold used during ASReview-assisted title-and-abstract screening and the proportion of extracted studies selected for independent verification. We have clarified and revised these procedures separately. For title-and-abstract screening, the 10% threshold did not mean that only 10% of records were screened. Records were divided into seven assigned screening sets, each assessed independently by two reviewers. Screening proceeded in the order prioritised by ASReview until reviewers encountered an uninterrupted sequence of irrelevant records equivalent to 10% of the records assigned to that set. Across the seven screening sets, this corresponded to 355–822 consecutive irrelevant records, depending on set size. One reviewer participated across all seven screening sets to support consistent application of the eligibility criteria, while the second reviewer differed between assigned sets. The 10% threshold was used as a pragmatic, set-size-proportional operational heuristic rather than as a validated or universally applicable stopping criterion. We have therefore removed wording suggesting that it represents an established or recommended stopping rule. ASReview was used only to prioritise the order in which records were presented; eligibility decisions remained with the reviewers. We also acknowledge that this approach cannot guarantee complete recall and that potentially eligible records may remain among lower-ranked unscreened records. The Methods – Selecting and appraising documents section has been revised on page 14, lines 299–309 to state: *"Records were divided into seven assigned screening sets, each assessed independently by two reviewers. One reviewer participated across all seven sets to support consistent application of the eligibility criteria, while the second reviewer differed between assigned sets. Records were screened iteratively in the order prioritised by ASReview. Within each set, screening continued until reviewers encountered an uninterrupted sequence of irrelevant records equivalent to 10% of the records assigned to that set. Across the seven screening sets, this corresponded to 355–822 consecutive irrelevant records, depending on set size. This set-size-proportional threshold was used as a pragmatic operational stopping heuristic informed by approaches to active-learning-assisted screening, rather than as a universally validated cut-off [39]. ASReview prioritised the presentation of records, but all eligibility judgements were made by the reviewers."* The corresponding limitation has been added to the Discussion – limitations paragraph on page 22, lines 499–503: *"In addition, the pragmatic stopping threshold used during ASReview-assisted title-and-abstract screening may have left some eligible lower-ranked records unscreened, because active learning prioritises records according to predicted relevance rather than guaranteeing that all potentially eligible records are encountered before screening stops [38,39]."* We also agree with the Reviewer that the originally planned independent verification of 10% of extracted studies was insufficiently justified. We have therefore increased the planned independent verification to 20%. The Methods – Data extraction and synthesis, subsection 'Organisation and classification of extracted data' has been revised on page 17, lines 378–384 to state: *"To assess the consistency of data extraction, a second reviewer will independently re-extract data from 20% of the included studies, selected to include both flood- and heat-related studies and the main study-design categories. Discrepancies will be discussed and resolved*

before synthesis, and the extraction guidance will be revised where necessary. If recurring or systematic discrepancies are identified, independent verification will be extended to additional studies and relevant data fields."

1.9 The article sells itself as a realist informed review but explicitly stating which elements have been adopted and which haven't and why would be helpful for the reader.

Response:

We thank Reviewer #1 for this important comment. We agree that the submitted manuscript did not define "realist-informed" clearly enough or distinguish the realist elements adopted in this review from those associated with a full realist review. We have therefore revised the Methods – Analytical framework section to make this distinction explicit. In this review, realist principles are used to structure the extraction, interpretation and synthesis of evidence on context, mechanisms and outcomes. For each reported health-service outcome, we will describe narratively the relevant climatic, hazard and health-system context, the intervention and the changes it introduces, and the mechanism reported by the study authors or, where supported by the available evidence, cautiously inferred by the review team. Climatic and hazard context will include Köppen–Geiger climate classification and, where reported, characteristics of the flood or heat event, such as hazard type, temperature or intensity, magnitude, duration and timing. We will then compare these C–M–O-informed relationships across intervention categories and settings to identify recurring, contrasting and disconfirming explanatory patterns. This approach directly supports the review objective of examining how intervention mechanisms and contextual factors may shape effects on MCH service delivery and utilisation. The review does not develop or test formal C–M–O configurations or an overarching programme theory. It also does not use iterative theory-driven searching, purposive theoretical sampling or realist relevance-and-rigour appraisal. These elements were not adopted because the review was designed to systematically identify and classify empirical evidence across multiple types of adaptation intervention and to examine mechanisms and context across this heterogeneous evidence base, rather than to develop and refine a single programme theory. Searching and study selection followed predefined systematic-review procedures, and methodological quality will be assessed using the Mixed Methods Appraisal Tool (MMAT) according to study design. We have also clarified the distinction between intervention characteristics and mechanisms. Intervention inputs, design features, implementation activities and health-system functions will not themselves be treated as mechanisms. In this review, a mechanism is understood as an underlying response or causal process through which an intervention may contribute to a health-service outcome in a particular context. Where a mechanism is not explicitly reported by study authors, it will be inferred cautiously only where the available evidence supports a plausible explanatory process; an association between an intervention and an outcome alone will not be considered sufficient evidence of a mechanism. The WHO climate-resilient health-system framework and Meadows' leverage-points framework will be used as complementary analytical frameworks rather than as components of realist methodology. The WHO framework will be used to identify where interventions operate within the health system and the vulnerabilities they address, while Meadows' framework will characterise the depth of system change represented by an intervention. Climatic and hazard characteristics, together with health-system, economic, geographic, social and governance conditions, will provide additional dimensions of context. Together, these dimensions will inform the C–M–O-informed narrative synthesis but will not themselves be treated as mechanisms. The Methods – Analytical framework section has been revised on pages 7–8, lines 171–184 to state: *"This review is realist-informed rather than a full realist review. In this review, 'realist-informed' means that C–M–O reasoning informs data*

extraction and will guide interpretation and synthesis, while searching and study selection followed predefined systematic-review procedures and methodological appraisal will be conducted using the design-specific criteria of the Mixed Methods Appraisal Tool (MMAT). The realist elements adopted are C–M–O-informed data extraction, cross-study comparison and explanatory narrative synthesis. Together, these frameworks will be used to examine how interventions function in practice, the mechanisms they trigger, and the parts of the health system they strengthen. Interventions will be grouped into thematic areas based on intervention type and the system vulnerabilities they address and then mapped against the ten components of the WHO framework and the twelve leverage points described by Meadows [24]. The vulnerabilities considered in this review are those identified in a previous systematic review of flood and heat impacts on the delivery of and access to maternal and child healthcare and are summarised in the "Vulnerability Addressed" domain of Figure 1 [11]. *The structured extraction template records intervention characteristics and functions, contextual factors, reported or cautiously inferred mechanisms, outcomes, barriers, enablers, policy support, co-benefits and disbenefits in separate fields. Contextual factors include climatic and hazard characteristics, health-system, economic, geographic, social and governance conditions. Climatic and hazard context will include Köppen–Geiger climate classification and, where reported, characteristics of the flood or heat event, such as hazard type, temperature or intensity, magnitude, duration and timing. In this review, a mechanism is understood as an underlying response or causal process through which an intervention may contribute to a health-service outcome in a particular context. Intervention inputs, design features, implementation activities and health-system functions will not themselves be treated as mechanisms. For each reported health-service outcome, the synthesis will describe narratively the relevant context, the intervention and the changes it introduces, and the mechanism reported by study authors or, where supported by the available evidence, cautiously inferred by the review team. Inferred mechanisms will be distinguished from author-reported mechanisms, and an association between an intervention and an outcome alone will not be considered sufficient evidence of a mechanism. These C–M–O-informed relationships will be compared within and across intervention categories and settings to identify recurring, contrasting and disconfirming explanatory patterns.* Study settings will also be classified by country income group, health-system context, including degree of health-sector decentralisation and universal health coverage status, and Köppen–Geiger climate zone [28,29]. *These classifications will be used to describe and compare the climatic and health-system contexts in which interventions have been reported. For interventions whose implementation or performance may be sensitive to climatic conditions, projected changes in Köppen–Geiger classification will be considered descriptively to identify possible climate analogues. A possible climate analogue will be defined as a setting whose projected future climate classification becomes similar to the present-day classification of a setting in which a comparable intervention has previously been reported with favourable health-service delivery or utilisation outcomes [29–31]. The identification of a possible climate analogue will be treated as an exploratory, hypothesis-generating signal that an intervention may warrant further context-specific assessment. It will not be interpreted as evidence that the intervention is transferable or will produce comparable outcomes in the future setting. Climatic similarity will be considered alongside country income, health-system organisation and capacity, governance, infrastructure, workforce availability, service access, geographic and social conditions, intervention design and implementation requirements. Climate-zone similarity will therefore be treated as one dimension of context rather than as a sufficient basis for recommending the transfer of an intervention. The WHO climate-resilient health-system framework and Meadows' leverage-points framework provide complementary analytical structures. The WHO framework will be used to identify where interventions operate within the*

health system and the vulnerabilities they address, while Meadows' framework will be used to characterise the depth of system change represented by an intervention, ranging from relatively limited adjustments to system inputs and parameters to broader changes in system goals, structures or power relations [24,25]. These frameworks and classifications support the C–M–O-informed synthesis but will not themselves be treated as mechanisms or as components of realist methodology."

Although we do not pre-specify context mechanism outcome configurations, as the review will identify a range of intervention categories which may target distinct outcomes, we draw on realist principles, to synthesize evidence on why change associated with specific interventions occurs in some contexts and not others and the health system mechanisms by which they occur (Lokmic-Tomkins et al., 2023; Richmond et al., 2024; Schwerdtle et al., 2024). *The review does not develop or test formal C–M–O configurations or an overarching programme theory and does not use iterative theory-driven searching, purposive theoretical sampling or realist relevance-and-rigour appraisal. These elements were not adopted because the review was designed to identify and classify empirical evidence across multiple intervention categories and to examine mechanisms and contextual influences across this heterogeneous evidence base rather than to develop and refine a single predefined programme theory. A predefined systematic search was therefore used, and methodological quality will be assessed using the Mixed Methods Appraisal Tool (MMAT) to provide a consistent, design-specific appraisal of qualitative, quantitative and mixed-methods studies (Hong et al., 2018). Realist reasoning will guide interpretation and synthesis but will not replace the systematic procedures used to identify, select and appraise the evidence."*

1.10 Climate zone transferability claims are undoubtably very exciting but perhaps could be toned down somewhat as there are inherent differences between different regions not only related to the climate so perhaps the exploratory nature could be emphasised more?

Response:

We thank Reviewer #1 for this important comment. We agree that the wording in the submitted manuscript could be interpreted as suggesting that similarity between climate zones is sufficient to establish the transferability of an intervention. This was not our intention. We have therefore toned down these claims in the relevant sections of the manuscript and now describe the proposed climate-analogue assessment explicitly as exploratory and hypothesis-generating. We have clarified that Köppen–Geiger climate classification represents only one dimension of context. Climatic similarity will be considered alongside differences in health-system organisation and capacity, governance, financing, infrastructure, workforce availability, service access, population needs, geographic and social conditions, and intervention design and implementation. A possible climate analogue will therefore be treated only as an indication that an intervention may warrant further context-specific assessment, rather than as evidence that it is transferable or will produce comparable outcomes in another setting. We have revised the Abstract – Methods and analysis, Methods – Analytical framework, and Discussion – limitations accordingly. The Abstract – Methods and analysis section has been revised on page 2, lines 34–37 to state: *"Study settings will be classified by country income group, health-system context and Köppen–Geiger climate zone to compare evidence across settings and, where appropriate, to identify possible climate analogues for exploratory, context-specific assessment. Climate-zone similarity will not be used to infer intervention transferability or future effectiveness."* The Methods – Analytical framework section has been revised on page 9, lines 205–220 to state:

"These classifications will be used to describe and compare the climatic and health-system contexts in which interventions have been reported. For interventions whose implementation or performance may be sensitive to climatic conditions, projected changes in Köppen–Geiger classification will be considered descriptively to identify possible climate analogues. A possible climate analogue will be defined as a setting whose projected future climate classification becomes similar to the present-day classification of a setting in which a comparable intervention has previously been reported with favourable health-service delivery or utilisation outcomes [29–31]. The identification of a possible climate analogue will be treated as an exploratory, hypothesis-generating signal that an intervention may warrant further context-specific assessment. It will not be interpreted as evidence that the intervention is transferable or will produce comparable outcomes in the future setting. Climatic similarity will be considered alongside country income, health-system organisation and capacity, governance, infrastructure, workforce availability, service access, geographic and social conditions, intervention design and implementation requirements. Climate-zone similarity will therefore be treated as one dimension of context rather than as a sufficient basis for recommending the transfer of an intervention." The Discussion – limitations section has been revised on pages 22–23, lines 515–533 to state: *"The Köppen–Geiger classification provides a standardised way of describing climatic context but cannot by itself establish whether an intervention is transferable between settings [28,29]. Comparisons across climate and health-system settings will therefore be exploratory, as even when climate zones and health-system settings are comparable, this does not necessarily mean that intervention effects will be the same, as implementation and other contextual factors can moderate intervention effects. Locations with similar climatic classifications may differ substantially in hazard characteristics, health-system organisation and capacity, governance, financing, infrastructure, workforce availability, service access, population needs and implementation conditions. The climate-analogue assessment will therefore be exploratory and hypothesis-generating, identifying interventions that may warrant further context-specific assessment rather than establishing transferability or predicting future effectiveness [29–31]. C–M–O-informed relationships and explanatory patterns will likewise be compared across, rather than generalised across, intervention categories and settings, and interpreted as context-dependent explanations of how interventions may work under particular conditions rather than as evidence that the same intervention will necessarily produce the same outcomes elsewhere [26,27]. These limitations mean that the review findings should be interpreted as identifying interventions and C–M–O-informed explanatory patterns that warrant further context-specific assessment, rather than as definitive evidence that an intervention will be effective in another setting."*

Reviewer #2

2.1 This manuscript presents a well-conceived protocol for a realist-informed systematic review on health-system interventions designed to protect routine maternal and child health (MCH) services during floods and heatwaves. The topic is highly timely and policy-relevant, given the accelerating impacts of climate change on health systems globally. The authors have developed a sophisticated analytical framework that integrates the WHO climate-resilient health systems building blocks, Meadows' leverage points, and realist C-M-O reasoning—a novel and theoretically grounded approach. The search strategy is comprehensive, covering eight databases with a well-documented search string. The use

of ASReview for active-learning-assisted screening and dual-independent screening adds methodological rigor. However, there are some problems with this manuscript.

Response:

We appreciate Reviewer #2's careful assessment of the protocol and recognition of the relevance of the review, the integrated analytical framework, the breadth of the search strategy and the screening procedures. We have carefully considered the concerns raised and revised the manuscript accordingly. Detailed responses and the corresponding manuscript changes are provided under Reviewer #2, Comments 2.4–2.8.

2.2 Due to the great meaning and a possibility in an improvement after a major revision, I suggest the editor accept this manuscript with major revision.

Response:

We appreciate Reviewer #2's positive recommendation and the opportunity to revise the manuscript. We have carefully addressed each concern and made substantial revisions to improve the clarity, transparency and methodological precision of the protocol.

2.3 I have some comments and suggestions for this manuscript as follows:

Response:

We appreciate Reviewer #2's comments and suggestions. Each substantive comment is reproduced and addressed separately below.

2.4 The authors state that screening will stop once reviewers encounter a consecutive run of irrelevant records equivalent to 10% of records assigned to that set (page 13, lines 247–249). This is a pragmatic heuristic, but it is not a standardly validated stopping criterion in active-learning-assisted screening. Empirical studies on ASReview have shown that stopping rules vary widely, and a fixed percentage may either miss eligible records or lead to premature termination, depending on the prevalence of relevant records in the set.

Response:

We thank Reviewer #2 for raising this concern. This is closely related to the issue raised by Reviewer #1, Comment 1.8, and the full rationale and corresponding manuscript revisions are provided in our response to that comment. We agree that an uninterrupted sequence of irrelevant records equivalent to 10% of a screening set is not a standardised or universally validated stopping criterion for ASReview-assisted screening. We have therefore revised the manuscript to describe this threshold explicitly as a pragmatic, set-size-proportional operational heuristic and have removed wording suggesting that it represents an established or recommended stopping rule. The threshold was applied separately to each of the seven screening sets, meaning that the required number of consecutive irrelevant records varied according to set size. Across the seven sets, this corresponded to 355–822 consecutive irrelevant records. The use of a proportional threshold does not, however, guarantee complete recall. We therefore acknowledge that potentially eligible studies may remain among lower-ranked unscreened records. We have also clarified that ASReview was used only to prioritise the order in which records were presented; all eligibility decisions were made independently by the reviewers. The corresponding limitation is now stated explicitly in the Discussion. To avoid duplicating the same revised manuscript text, the complete wording added to the Methods – Selecting and appraising documents section and the Discussion – limitations paragraph is reproduced under Reviewer #1, Comment #1.8.

2.5 The protocol states that C-M-O configurations will be developed, but the methods for deriving mechanisms from the included studies are not fully described. Realist

reviews typically involve retroduction—inferring underlying mechanisms from patterns observed across studies. The protocol currently describes data extraction and classification but does not explain how the team will move from extracted data to C-M-O configurations.

Response

We thank Reviewer #2 for highlighting this lack of clarity. We agree that the submitted manuscript gave the impression that the review would develop and refine formal C–M–O configurations without adequately describing how this would be done. As clarified in our response to Reviewer #1, Comment #1.9, this wording overstated the realist component of the review. We are not developing or testing formal C–M–O configurations. We have therefore revised the manuscript to describe the analysis more accurately as a C–M–O-informed narrative explanatory synthesis. Mechanism-related information will be considered separately from intervention characteristics and functions, barriers and enablers, and outcomes. In this review, a mechanism is understood as an underlying response or causal process through which an intervention may contribute to a health-service outcome in a particular context. Intervention inputs, design features, implementation activities and health-system functions will not themselves be treated as mechanisms. For each reported health-service outcome, we will describe narratively the relevant climatic, hazard and health-system context, the intervention and the changes it introduces, the mechanism reported by the study authors or, where supported by the available evidence, cautiously inferred by the review team, and the reported outcome. Mechanisms explicitly reported by study authors will be distinguished from those inferred by the review team. Where a mechanism is not stated directly, inference may draw on qualitative accounts, implementation findings, temporal relationships, differences between settings or population groups, barriers and enabling conditions, and study authors' explanations of why an intervention succeeded, failed or produced different outcomes. An observed association between an intervention and an outcome alone will not be considered sufficient evidence of a mechanism. These C–M–O-informed relationships will then be compared within and across intervention categories, health-system vulnerabilities and climatic and health-system settings to identify recurring, contrasting and disconfirming explanatory patterns. Interpretation will take account of the design and methodological quality of the supporting studies. This approach is intended to examine how and why interventions may produce different outcomes under different contextual conditions, rather than to construct or test formal C–M–O configurations or an overarching programme theory. The WHO climate-resilient health-system framework and Meadows' leverage-points framework will provide complementary classifications: the WHO framework will identify where an intervention operates within the health system and the vulnerabilities it addresses, while Meadows' framework will characterise the depth of system change represented by the intervention. These frameworks support classification and interpretation but will not themselves be treated as mechanisms or as C–M–O components. The Methods – Data extraction and synthesis, subsection (4) Data analysis and synthesis has been revised on pages 19–20, lines 421–447 to state: *"To support the realist-informed synthesis, mechanism-related information will be considered separately from intervention characteristics and functions, barriers and enablers, and outcomes. In this review, a mechanism is understood as an underlying response or causal process through which an intervention may contribute to a health-service outcome in a particular context. Intervention inputs, design features, implementation activities and health-system functions will not themselves be treated as mechanisms. For each reported health-service outcome, the synthesis will describe narratively the relevant climatic, hazard and health-system context, the intervention and the changes it introduces, the mechanism reported by study authors or, where supported by the available evidence, cautiously inferred by the review team, and the reported outcome. Mechanisms explicitly reported by study authors will be distinguished from those inferred by the review*

team. Where a mechanism is not stated directly, inference may draw on qualitative accounts, implementation findings, temporal relationships, differences between settings or population groups, barriers and enabling conditions, and explanations provided in the study for why an intervention succeeded, failed or produced different outcomes. An association between an intervention and an outcome alone will not be considered sufficient evidence of a mechanism. These C–M–O-informed relationships will be compared within and across intervention categories, health-system vulnerabilities and climatic and health-system settings to identify recurring, contrasting and disconfirming explanatory patterns. Interpretation will take account of the design and methodological quality of the supporting studies. The synthesis will therefore use C–M–O reasoning to explain how interventions may contribute to different outcomes under different contextual conditions, rather than developing or testing formal C–M–O configurations or an overarching programme theory. The WHO climate-resilient health-system framework will be used to identify where interventions operate within the health system and the vulnerabilities they address, while Meadows' leverage-points framework will be used to characterise the depth of system change represented by the intervention [24,25]. These frameworks will support classification and interpretation but will not themselves be treated as mechanisms."

2.6 The protocol includes quantitative descriptive studies (cross-sectional surveys, case series) alongside qualitative, RCT, and quasi-experimental studies. While this inclusivity is appropriate for a realist review, quantitative descriptive studies without comparison groups may provide limited evidence on intervention effects and may bias the synthesis if not critically appraised separately.

Response:

We thank Reviewer #2 for this important comment. We agree that quantitative descriptive studies without an appropriate comparison or evaluative structure provide more limited evidence on intervention effects than randomised controlled trials and appropriately designed quasi-experimental evaluations. We have therefore clarified both the range of evaluative study designs considered in the review and the distinct role of descriptive evidence in the synthesis. In addition to randomised controlled trials, relevant quasi-experimental designs may include natural experiments, interrupted time-series, controlled interrupted time-series, controlled before–after studies and other appropriate longitudinal comparative designs. We have also clarified that the absence of a separate contemporaneous control group does not necessarily mean that a study lacks an appropriate comparative or counterfactual structure. For example, interrupted time-series designs use repeated observations before and after an intervention and can estimate changes in level or trend relative to the pre-intervention trajectory. This distinction is particularly relevant to interventions implemented during floods, heatwaves and other extreme events, where the timing and unpredictability of the event, together with operational and ethical constraints, may make randomisation or conventional concurrent control groups difficult to implement. Quantitative descriptive studies remain eligible because they may provide intervention-specific evidence relevant to implementation, context, feasibility, barriers, enablers, reported service patterns and reported mechanisms or evidence relevant to cautiously inferred mechanisms. We considered excluding non-comparative descriptive studies entirely; however, because the review also examines intervention implementation, context and mechanisms, we have retained those that provide relevant intervention-specific evidence in these domains, while excluding them from conclusions about intervention effectiveness**.** Non-comparative descriptive studies will therefore not be used to estimate or attribute intervention

effects or to establish intervention effectiveness. Their contribution to the synthesis will be restricted to explanatory and implementation-related domains, and they will be presented and interpreted separately from evaluative evidence. This distinction is intended to prevent descriptive evidence from being given the same inferential weight as appropriately designed randomised or quasi-experimental evaluations. The extraction template records study design separately from comparator use. Comparator use is recorded using predefined categories distinguishing studies with a control-group comparison, studies reporting a pre–post comparison, studies without a comparator and studies for which comparator information is not reported. The template also records direction of impact, reported change (Δ) and any reported effect size. These fields will be interpreted together with the underlying study design so that, for example, an interrupted time-series or other longitudinal quasi-experimental study is not considered weak evidence solely because it lacks a separate contemporaneous control group. Each included study will be critically appraised using the MMAT criteria appropriate to its design. Quantitative descriptive studies will therefore be appraised separately from randomised, non-randomised, qualitative and mixed-methods studies. The strength of any inference about intervention effects will take account of the study design, its comparative or counterfactual structure and methodological quality. The Methods – Data extraction and synthesis, subsection 'Data extraction' has been revised on page 16, lines 352–361 to state: *"The extraction framework records study design, comparator use, direction of impact, reported change and any reported effect size separately. Comparator use is recorded as a control-group comparison, a pre–post comparison, no comparator or not reported. Direction of impact is recorded as positive, neutral, negative, mixed or not reported, while the change field records a reported delta, change from baseline, before–after difference, no change or not reported. These fields will be interpreted together with the underlying study design. The absence of a separate contemporaneous control group will not by itself determine the strength of evidence, as some quasi-experimental approaches, including interrupted time-series designs, use repeated observations and the pre-intervention outcome trajectory to provide a counterfactual comparison."* The Methods – Data extraction and synthesis, subsection 'Quality and risk-of-bias assessment' has been revised on pages 17–18, lines 392–397 to state: *"Each study will be assessed using the MMAT criteria relevant to its design. Quantitative descriptive studies will therefore be appraised separately from randomised, non-randomised, qualitative and mixed-methods studies. The strength of any inference about intervention effects will be qualified according to study design, the comparative or counterfactual structure of the evaluation, and the relevant MMAT assessment."* The Methods – Data extraction and synthesis, subsection 'Data analysis and synthesis' has been revised on pages 18–19, lines 406–420 to state: *"Evidence will be interpreted according to study design, the comparative or counterfactual structure of the evaluation, and methodological quality. Randomised controlled trials and appropriately designed quasi-experimental evaluations, including natural experiments, interrupted time-series, controlled interrupted time-series, controlled before–after studies and other appropriate longitudinal comparative designs, will provide stronger evidence when assessing intervention effects than non-comparative descriptive studies. A separate contemporaneous control group will not be required for a study to contribute evaluative evidence where an appropriate quasi-experimental design provides a credible counterfactual. Quantitative descriptive and other non-comparative studies may contribute*

evidence on intervention implementation, context, feasibility, barriers, enablers, reported service patterns and reported mechanisms or evidence relevant to cautiously inferred mechanisms, but they will not be used to attribute an outcome to the intervention or establish intervention effectiveness. Evaluative and non-comparative descriptive evidence will be presented and interpreted separately in the evidence tables and narrative synthesis and will not be treated as methodologically equivalent evidence of intervention effectiveness." The corresponding limitation has also been added to the Discussion – limitations paragraph on page 22, lines 503–514: *"Differences in intervention types, study designs, outcome measures and settings may also limit direct comparisons between studies. Rigorous outcome evaluation may be challenging for interventions implemented during floods, heatwaves and other extreme events, where randomisation or conventional concurrent control groups may not always be feasible. Appropriately designed quasi-experimental approaches, including natural experiments and interrupted time-series designs, may therefore provide important evaluative evidence. Quantitative descriptive and other non-comparative studies may provide useful evidence on implementation, context and reported service changes but provide more limited evidence about intervention effects. They will not be used to attribute an outcome to an intervention or establish intervention effectiveness. Findings will therefore be interpreted according to study design, comparative or counterfactual structure and methodological quality rather than treating all forms of evidence as equivalent."*

2.7 The protocol states that "at the time of writing, the search, deduplication, title and abstract screening, full-text assessment, and data extraction had been completed" (page 8, lines 158–160). This is unusual for a protocol submission and raises questions about whether the review is truly prospective. While retrospective protocols are not necessarily disallowed, transparency about what has already been done is critical.

Response:

We thank Reviewer #2 for raising this important concern. We agree that the wording in the submitted manuscript was inaccurate because it suggested that all stages of the review, including screening, full-text assessment and data extraction, had already been completed. This was not the case, and we have removed this statement. As clarified in our responses to Reviewer #1, Comments #1.4 and #1.5, the initial database search covered evidence available up to 16 May 2025. The search was subsequently updated on 30 April 2026 to identify additional records published or indexed between 17 May 2025 and 30 April 2026. At the time of the original submission to BMJ Open on 9 June 2026, title-and-abstract screening, full-text assessment and data extraction for records identified through the updated search were ongoing; these activities remain ongoing at the time of this revision. Data extraction across the full review evidence base, including studies identified through both the initial and updated searches, also remains ongoing. Methodological appraisal, C–M–O-informed synthesis, integration of the analytical frameworks, final synthesis and reporting are planned. The protocol reports no review findings, analytical results or conclusions. The protocol manuscript was developed alongside the review activities. As explained more fully under Reviewer #1, Comment #1.5, it was initially submitted to BMJ Global Health on 1 May 2026 and subsequently transferred to BMJ Open after we were advised on 5 June 2026 that BMJ Global Health did not publish research protocols. Review activities therefore continued while the protocol was undergoing submission and transfer. We have revised the Methods – Study design section to distinguish clearly between the completed database searches and initial-search

screening procedures and the updated-search screening, full-text assessment and review-wide data extraction activities that remain ongoing. The complete revised Study design wording is reproduced under Reviewer #1, Comment #1.5. The separate issue of prospective registration is addressed under Reviewer #1, Comment #1.6, and Editor Comment #2.

2.8 Some recent study found significant associations of increased temperature or extreme heat with maternal and childhood chronic outcomes (e.g., allergic diseases, metabolic diseases) and infectious diseases (pneumonia, otitis media, COVID-19) (; https://doi.org/10.1016/j.buildenv.2024.112317; https://doi.org/10.1016/j.buildenv.2023.110668; https://doi.org/10.1016/j.buildenv.2023.110800). Furthermore, some other new studies explain the potential mechanisms underlying the associations, such as the expressions of transient receptor potential (TRP) ion channels (TRPV1, TRPV4, TRPA1, TRPM2) (https://doi.org/10.1016/j.jhazmat.2026.142315; https://doi.org/10.1016/j.envpol.2025.126321; https://doi.org/10.1111/all.15883). I suggest the authors to discuss your findings with (some of) the new articles and to further support your findings and discuss the potential mechanism to support your results.

Response:

We thank Reviewer #2 for drawing our attention to these recent studies. We reviewed all six cited articles and considered both their relevance to the Background and their eligibility for inclusion in the systematic review. The present manuscript is a protocol and does not report review findings or results. It is therefore not yet possible to discuss the findings of this review in relation to the suggested studies. We have instead incorporated the studies that are relevant to the rationale for the review into the Background, and relevant contextual evidence will be considered when interpreting the findings of the completed systematic review. Three of the suggested articles are human epidemiological studies examining associations between prenatal or early-life exposure to temperature or other meteorological conditions and childhood allergic diseases, asthma and pneumonia [6 - 7]. These studies are relevant to the demand-side rationale for the review because they indicate that climate-related exposures may contribute to childhood morbidity and consequently increase the need for healthcare. However, they did not directly assess health-service delivery or utilisation. We have therefore been careful not to infer increased healthcare utilisation from these studies. Although [6] examined household air-conditioning as a modifier of the association between temperature exposure and childhood allergic disease, it did not evaluate air-conditioning as an intervention intended to maintain or improve health-service delivery, access or utilisation. Similarly, the two studies [7, 8] did not evaluate eligible health-system adaptation interventions or report outcomes relating to health-service delivery, utilisation, coverage, continuity of care or health-system resilience. These three studies therefore provide relevant contextual evidence for the Background but do not meet the full eligibility criteria for the intervention synthesis. The remaining three suggested articles are experimental studies using mouse models to investigate biological or pathophysiological responses to extreme-temperature exposure, including oxidative stress, inflammation, transient receptor potential ion-channel activity and metabolic or cellular pathways. These studies provide evidence concerning the biological plausibility of temperature-related health effects but do not evaluate health-system adaptation interventions or health-service outcomes. Their biological mechanisms are therefore conceptually different from the health-system intervention mechanisms examined in this realist-informed review. As clarified in our responses to Reviewer #1, Comment #1.9, and Reviewer #2, Comment #2.5, this review uses C–M–O-informed reasoning to examine how an intervention may contribute to a health-service outcome under particular climatic and health-system conditions. Biological or pathophysiological pathways through which heat exposure contributes to disease are outside

this definition of an intervention mechanism. The Background has been revised on page 4, lines 82–92 to state: *"Climate-related hazards can affect MCH systems through interacting demand- and supply-side pathways. On the demand side, recent epidemiological studies have reported associations between prenatal or early-life exposure to ambient temperature and other meteorological conditions and childhood allergic diseases, asthma and pneumonia [6–8]. Although these studies did not examine health-service delivery or utilisation, their findings indicate that climate-related exposures may increase childhood morbidity and consequently the need for healthcare. On the supply side, floods can damage healthcare infrastructure, interrupt medical supply chains and cause power outages, while heat can disrupt cold chains and medicine storage, reduce workforce capacity and worsen patient care experiences. An increase in healthcare need may therefore occur at the same time that floods and heatwaves reduce the capacity of health systems to provide accessible and continuous care."* The distinction between biological pathways and the mechanisms examined in this review has also been clarified in the Methods – Analytical framework, as detailed in our responses to Reviewer #1, Comment #1.9, and Reviewer #2, Comment #2.5. The three human epidemiological studies were therefore added to the Background because they strengthen the demand-side rationale for the review. The three experimental animal studies were considered but were not added because their biological mechanisms do not directly inform the health-system intervention focus of this protocol. None of the six suggested studies meets the full eligibility criteria for inclusion in the intervention synthesis.
