# Supplementary material for: Health system interventions to floods and heatwaves for maternal and child health services: a realist-informed systematic review protocol
Source: BMJ Open. 2026 Sep 11;16(9):e123914. doi: 10.1136/bmjopen-2026-123914 (PMC13583685; doi:10.1136/bmjopen-2026-123914)
Supplement: Supplementary file 2 [file bmjopen-16-9-s001.docx]

**Supplementary Information (SI)**

**For**

**Health-system interventions to floods and heatwaves for maternal and child health services: a realist-informed systematic review protocol**

Sisay E. Debele¹^,^²^[1]^, Fiammetta Bozzani¹, Grace Mushinda-Musonda³, Sari Kovats4, Gabrielle Bonnet¹, Chitalu Chama-Chiliba³, Anna Foss¹, Everton Nunes Da Silva^4^**,** Josephine Borghi¹^,^⁵

*¹Department of Global Health and Development, Faculty of Public Health and Policy, London School of Hygiene & Tropical Medicine, London WC1H 9SH, United Kingdom*

*²Institute of Disaster Risk Management and Food Security Studies, Bahir Dar University, P.O. Box 5501, Bahir Dar, Ethiopia³ Centre for Economic and Social Research, University of Zambia, P.O. Box 30900, Lusaka 10101, Zambia*

*^4^Department of Public Health, Environments and Society, Faculty of Public Health and Policy, London School of Hygiene & Tropical Medicine, London WC1H 9SH, United Kingdom*

*^5^Faculty of Ceilândia, University of Brasília, Brasília, Brazil*

*^6^International Institute for Applied Systems Analysis (IIASA), Laxenburg, Austria*

**Table S1**. Summary of objective/aim past 17 review papers on interventions to increase adaptation and resilience of health systems to climate change extremes.

| **Location** | **Intervention** | **Summary of the aims/objectives** | **Authors (Year)** |
| --- | --- | --- | --- |
| Global | - Climate change integration into health policies - Investment in climate-resilient technologies and infrastructure - Capacity building for climate Specialists and Agencies | This review aims to support the development of robust, context-specific policies for building climate-resilient health systems and the communities they serve. It focuses on providing insights into efficient, cost-effective solutions for addressing adaptation challenges in developing countries, aligning with the WHO’s resilience framework to ensure continuity of essential health services during climate-related crises. The review is guided by two research questions: (1) What are the health system adaptation strategies to climate change? and (2) What are the barriers to effective health system responses for climate change adaptation? | *^2^Ansah et al. (2024)* |
| Global | - Health system strengthening - National adaptation plans for health - Early warning systems - Policy integration and Coherence | Through a comprehensive examination of relevant literature, this review explores the current understanding of the connection between Disaster Risk Reduction (DRR) and Climate Change Adaptation (CCA) in health. It highlights opportunities to enhance conceptual synergies, such as fostering resilience and minimizing vulnerability and risk. | *^3^Banwell et al. (2018)* |
| Global | - Cooling Roofs - Mental health screening and Support | This paper examines the evolution of vulnerability and adaptation (V&A) assessments as tools for governments and experts to address climate change risks to health. It reviews the guidance developed for conducting V&As and summarizes national and subnational assessments conducted to date. The paper underscores the role of these assessments in building the global evidence base on climate change impacts on health and highlights the use of findings to advance health adaptation efforts. It concludes with recommendations for future V&As, establishing baselines of climate-related health risks and adaptation activities, and applying this knowledge to safeguard population health. | *^3^Berry et al. (2018)* |
| Fiji, Papua New Guinea | - Village relocation - Early warning systems - Integrated water resource management - Health national adaptation plans - Managed retreat | The study synthesized research on climate change and human health in the Pacific to address three key questions: how health risks of climate change and associated policy responses compare across Pacific Island Countries (PICs), what knowledge gaps exist in the region, and what evidence-based approaches can support policymaking to effectively manage current and emerging climate-related health impacts. | *^3^Bowen et al. (2024)* |
| Benin | - Health System Strengthening - Policy and Planning - Financing and Implementation - Information and Capacity Building - Societal Resilience - Disaster Risk Prevention - Preparedness, Response, and Recovery - Mitigation | The aim of this study was to review the literature on health system adaptation in sub-Saharan Africa | *^2^**Hounkpatin et al. (2024)* |
| Global | - Heat-Health Action Plans - Heat-Health Warning Systems - Urban Planning and Development Plans - Real-Time Surveillance and Monitoring Systems - Dynamic Alert Systems | This study focused on several key objectives: reviewing the genesis, historical timeline, and definitions of Heat Action Plans (HAPs) and Health Heat Action Plans (HHAPs); analysing thresholds, heatwave measurement, and the scale of HAP/HHAP implementation; exploring the content and context of these plans across spatial scales; identifying issues, concerns, and gaps in existing heat response plans; investigating the integration of HAPs/HHAPs with urban planning policies and development plans; and examining the potential of spatial frameworks to evaluate and mitigate heat vulnerability. | *^1^Kotharkar et al. (2022)* |
| Global | - Individual Interventions - Community-Based Interventions - Public Health Interventions - Health System Interventions - Policy Interventions - Health Promotion Programs - Health Protection Strategies - Climate Change Adaptation Strategies | This scoping review aims to map the evidence on the types, characteristics, and outcomes of multilevel interventions implemented as adaptive strategies to protect (MCH from the adverse effects of climate change. Two research questions were defined through iterative consultation with the research team: (1) What interventions are implemented at individual, community, and policy levels to protect MCH in response to climate change? and (2) What information exists about the interventions' descriptions, cultural contexts of design or implementation, and potential sustainability? | *^2^Lokmic-Tomkins et al. (2023)* |
| South Africa and Ghana | - Renewable Energy Systems - Health Workforce Education - Primary Health Care (PHC) Infrastructure Resilience - Health Information System Indicators - Multisectoral Collaboration - Community-Oriented Primary Health Care (PHC) | The review aimed to assess the scope of literature on climate change and primary health care in the African context and to identify knowledge gaps that could guide future research agendas. | *^2^Lokotola et al. (2023)* |
| Global | - Water Management - Wastewater Management - Waste Management - Energy Management - Chemical Waste Management - Green Purchasing Policy - Leadership - Green Building Design - Technological Advancements - Education and Research - Advocacy | This study aimed to explore the components of hospital adaptation to climate change and review the measures implemented to address climate change within healthcare facilities. | *^1^**Mashallahi et al. (2022)* |
| Global | - Strong Governance and Leadership - Raising Public Awareness - Strategic Resource Allocation - Climate Change Mitigation - Emergency Preparedness - Robust Health Service Delivery - Supporting Research | This study sought to identify interventions and actions that can enhance the resilience of health systems to effectively address the adverse health impacts of climate change. | *^2^Mosadeghrad et al. (2023)* |
| Bangladesh | - Child Immunization - Oral Rehydration Therapy - Vitamin A Supplementation - Family Planning Programs - Sector-Wide Approach under 4th HPNSP - Health National Adaptation Plan - 5-Point Scale for Climate Change Integration in Operational Plans | Health adaptation to climate change is a critical issue in Bangladesh but remains underexplored from the perspectives of health systems and policy environments. To address this gap and provide a comprehensive understanding of the situation, this scoping review examines the impacts of climate change on population health in Bangladesh and evaluates the policy environment and health system preparedness to tackle these challenges. | *^2^Nahian et al. (2023)* |
| ICs and LMICs | - Energy Efficiency - Reusable Equipment - Anaesthetic Management - Telemedicine and Mobile Outreaches - Infrastructure Resilience - Decentralized Surgical Services | This review examines how climate change can be integrated into national surgical planning in the Western Pacific region. | *^3^Qin et al. (2022)* |
| Philippines, India, and Thailand | - Community Outreach Campaigns - Workforce Training - Community Mobilization - Emergency Response Measures - Risk Monitoring and Disease Surveillance - Governance and Incident Command Systems - Medical Supply Management | This review aimed to complement conceptual analyses of health system resilience by providing greater specificity in research. Its objective was to map and describe how the existing literature has operationalized national health system resilience to severe extreme weather events in the Asia-Pacific region. | *^2^**Rameshshanker et al. (2021)* |
| LMICs | - Governance and Policy Activities - Vulnerability and Capacity Assessments (VCA) - Health Information and Early Warning Systems - Infrastructure and Technology Upgrades - Community Engagement - Climate-Informed Health Programs - Health Workforce Training | This scoping review aims to bridge top-down guidelines with practical bottom-up activities to drive transformational change in the health sector. | *^2^**Schwerdtle et al. (2024)* |
| Global | - Hazard Vulnerability Analysis - Climate-Resilient Infrastructure - Emergency Preparedness Protocols - Supply Chain Resilience Measures - Sustainable Energy Use - Health Care Emissions Mitigation | This review aims to synthesize evidence on how the healthcare sector can adapt to a changing climate while reducing its own climate impact. It seeks to identify barriers to implementing these adaptations and offers actionable recommendations for building sustainable and resilient healthcare systems. | *^3^Sherman et al. (2023)* |
| Global | - Transformational leadership - Emergency preparedness and planning - Core climate change competencies training - Multi-sectoral collaboration - Telehealth and digital health strategies - Occupational health and safety plans - Climate mitigation infrastructure | This review examines the impact of climate change on the health system workforce, encompassing both public health and healthcare service delivery, and explores adaptation strategies used to mitigate the effects of extreme climate events. By reviewing empirical literature, the study aims to provide recommendations to enhance occupational health and safety for the workforce, generate adaptation strategies to mitigate future risks based on past experiences, and better prepare the health system for an uncertain climate future. | *^4^**Tsakonas et al. (2024)* |
| Global | - Training/skill development - Workforce capacity planning - Interdisciplinary collaboration - Role flexibility and substitution - Psychological support programs - Flexible scheduling and deployment plans | To guide workforce planning, skilling, and capacity-building policies and programs that address the challenges of climate change, the study conducted a rapid review of peer-reviewed literature. The review aimed to answer two key questions: What are the impacts of climate-related events on the health workforce? and How are health systems preparing their workforces to respond to the impacts of climate change? | *^4^**Zurynski et al. (2024)* |

*^1^Systematic review; ^2^A scoping review; ^3^Narrative review; ^4^Rapid review.*

**Table S2.** Search strategies by database for systematic review on climate adaptation interventions for health systems.

| **Database** | **Final Search strategy** |
| --- | --- |
| Web of Science | TS=( ("climate change" OR flood* OR rain* OR "heat stress" OR "hot temperature*" OR "high temperature*" OR heatwave* OR "temperature rise" OR "extreme weather" OR drought* OR wildfire* OR "environmental temperature*" OR overheating OR humidity)  AND  (adapt* OR strateg* OR prepared* OR "risk reduction" OR "resilient infrastructure" OR "nature-based solution*" OR "ecosystem-based adaptation" OR "climate-proof*" OR "early warning system*" OR "emergency response" OR "disaster response" OR "climate resilience" OR mitigation)  AND  ("health system*" OR "health service*" OR hospital* OR clinic* OR "primary care" OR "healthcare access" OR "service delivery" OR "health infrastructure" OR "health workforce" OR "continuity of care" OR hospitalization OR coverage)) |
| Medline ALL (Ovid) | (flood*.mp. OR rain*.mp. OR heat*.mp. OR high temperature*.mp. OR hot temperature*.mp. OR overheating.mp. OR temperature change*.mp. OR temperature rise.mp. OR hot weather.mp. OR extreme temperature.mp. OR environmental temperature*.mp. OR ambient temperature.mp. OR exposure.mp. OR thermal discomfort.mp. OR humidity.mp. OR nighttime temperatures.mp. OR exp Hot Temperature/ OR exp Heat Stress Disorders/ OR exp Floods/)  AND  (adapt*.mp. OR risk reduction*.mp. OR hazard adaptation.mp. OR exp Emergency Preparedness/ OR prepared*.mp. OR evacuation strateg*.mp. OR contingency planning.mp. OR nature-based solution*.mp. OR ecosystem-based adaptation.mp. OR EbA.mp. OR green-blue infrastructure.mp. OR climate-proof*.mp. OR resilient infrastructure.mp. OR health emergency governance.mp. OR risk evaluation*.mp. OR emergency response.mp. OR disaster response.mp. OR exp Disaster Planning/ OR public health emergency plans.mp. OR risk management*.mp. OR crisis management.mp. OR exp Crisis Intervention/ OR action*.mp. OR measure*.mp. OR protection*.mp. OR planning*.mp. OR exp Health Planning/ OR initiative*.mp. OR policy*.mp. OR exp Health Policy/ OR adopt*.mp. OR adjust*.mp. OR acclimat*.mp. OR compatibl*.mp. OR cease.mp. OR tackle.mp. OR address.mp. OR fight.mp. OR strateg*.mp. OR effort*.mp. OR attempt*.mp. OR framework*.mp. OR plan*.mp. OR approach*.mp. OR response*.mp. OR system strengthen*.mp. OR health system resilience.mp. OR surge capacity planning.mp. OR early warning system*.mp. OR warning system*.mp. OR manage*.mp. OR risk governance.mp. OR financing.mp. OR exp Health Financing/ OR health workforce resilience.mp. OR occupational heat stress management.mp. OR capacity building.mp. OR exp Capacity Building/)  AND  (exp Health Services/ OR health service*.mp. OR exp Hospitals/ OR hospital*.mp. OR clinic*.mp. OR exp Primary Health Care/ OR primary care.mp. OR exp Health Facilities/ OR health facilit*.mp. OR exp Continuity of Patient Care/ OR continuity of care.mp. OR exp Delivery of Health Care/ OR service delivery.mp. OR exp Health Services Accessibility/ OR access to care.mp. OR demand.mp. OR exp Hospitalization/ OR hospitalization.mp. OR utilisation*.mp. OR utilization*.mp. OR coverage.mp. OR exp Maternal Health Services/ OR maternal health services.mp. OR exp Child Health Services/ OR child health services.mp. OR exp Health Workforce/ OR health workforce.mp. OR exp Occupational Diseases/pc OR occupational heat stress.mp.) |
| EMBASE (Ovid) | Embase Classic+Embase <1947 to 2025 May 15> (exp heat stress disorder/ or exp hot temperature/ or flood*.ti,ab. or climate change.ti,ab. or exp climate change/)  AND  (adapt* or strateg* or resilience or prepared*).ti,ab.  AND  (exp health care delivery/ or exp hospital/ or exp primary health care/ or health service*.ti,ab.) 1721 |
| Global Health (CAB Direct) | (Global Health <1910 to 2025 Week 20>((heat* or flood* or "climate change")  AND  (adapt* or strateg* or resilience or prepared*)  AND  ("health system*" or "health service*" or hospital* or "primary care")).mp. [mp=abstract, title, original title, broad terms, heading words, cabicodes words] |
| EconLit | Econlit <1886 to May 01, 2025> (("climate change" or "heat stress" or flood*) and (adapt* or strateg* or resilience) and ("health system*" or "health service*" or hospital* or "economic impact*")).ti,ab. ((flood* or rain* or heat* or high temperature* or hot temperature* or overheating or temperature change* or temperature rise* or hot weather* or extreme temperature* or environmental temperature* or ambient temperature* or exposure or thermal discomfort* or humidity or nighttime temperatures*)  AND  (adapt* or risk reduction* or hazard adaptation or prepared* or evacuation strateg* or contingency planning or nature-based solution* or ecosystem-based adaptation or EbA or green-blue infrastructure or climate-proof* or resilient infrastructure or health emergency governance or risk evaluation* or emergency response or disaster response or public health emergency plans or risk management* or crisis management or action* or measure* or protection* or planning* or initiative* or policy* or adopt* or adjust* or acclimat* or compatibl* or cease or tackle* or address or fight or strateg* or effort* or attempt* or framework* or plan* or approach* or response* or system strengthen* or health system resilience or surge capacity planning or early warning system* or warning system* or manage* or risk governance or financing or health workforce resilience or occupational heat stress management or capacity building)  AND  (health service* or hospital* or clinic* or primary care or health facilit* or continuity of care or service delivery or access to care or demand or hospitalization or utilisation* or utilization* or coverage or maternal health services or child health services or health workforce or occupational heat stress)).mp. |
| GreenFILE | flood* OR rain* OR heat* OR "high temperature*" OR "hot temperature*" OR overheating OR "temperature change*" OR "temperature rise" OR "hot weather" OR "extreme temperature*" OR "environmental temperature*" OR "ambient temperature" OR exposure OR "thermal discomfort" OR humidity OR "nighttime temperatures"  AND  adapt* OR "risk reduction*" OR "disaster risk reduction" OR DRR OR "hazard adaptation" OR "emergency preparedness" OR prepared* OR "evacuation strategy" OR "contingency planning" OR "nature-based solution*" OR "ecosystem-based adaptation" OR EbA OR "green-blue infrastructure" OR "climate-proof*" OR "resilient infrastructure" OR "flood-resistant hospital design" OR "health emergency governance" OR "risk evaluation*" OR "emergency response" OR "disaster response" OR "public health emergency plans" OR "risk management*" OR "crisis management" OR "disaster risk reduction in health systems" OR action* OR measure* OR protection* OR planning* OR initiative* OR policy* OR adopt* OR adjust* OR acclimat* OR compatibl* OR cease OR tackle OR address OR fight OR strateg* OR effort* OR attempt* OR framework* OR plan* OR approach* OR response* OR "system strengthen*" OR "health system resilience" OR "surge capacity planning" OR "emergency hospital response" OR "early warning system*" OR "extreme weather alert systems" OR "warning system*" OR manage* OR "risk governance" OR financing OR "health workforce resilience" OR "occupational heat stress management" OR "capacity building"  AND  "health system*" OR "health service*" OR hospital* OR clinic* OR "primary care" OR "health facilit*" OR "continuity of care" OR "service delivery" OR "access to care" OR demand OR hospitalization OR utilisation* OR utilization* OR coverage OR "maternal health services" OR "child health services" OR "health workforce" OR "occupational heat stress" |
| CINAHL Complete | flood* OR rain* OR heat* OR "high temperature*" OR "hot temperature*" OR overheating OR "temperature change*" OR "temperature rise" OR "hot weather" OR "extreme temperature*" OR "environmental temperature*" OR "ambient temperature" OR exposure OR "thermal discomfort" OR humidity OR "nighttime temperatures"  AND  adapt* OR "risk reduction*" OR "disaster risk reduction" OR DRR OR "hazard adaptation" OR "emergency preparedness" OR prepared* OR "evacuation strategy" OR "contingency planning" OR "nature-based solution*" OR "ecosystem-based adaptation" OR EbA OR "green-blue infrastructure" OR "climate-proof*" OR "resilient infrastructure" OR "flood-resistant hospital design" OR "health emergency governance" OR "risk evaluation*" OR "emergency response" OR "disaster response" OR "public health emergency plans" OR "risk management*" OR "crisis management" OR "disaster risk reduction in health systems" OR action* OR measure* OR protection* OR planning* OR initiative* OR policy* OR adopt* OR adjust* OR acclimat* OR compatibl* OR cease OR tackle OR address OR fight OR strateg* OR effort* OR attempt* OR framework* OR plan* OR approach* OR response* OR "system strengthen*" OR "health system resilience" OR "surge capacity planning" OR "emergency hospital response" OR "early warning system*" OR "extreme weather alert systems" OR "warning system*" OR manage* OR "risk governance" OR financing OR "health workforce resilience" OR "occupational heat stress management" OR "capacity building"  AND  "health system*" OR "health service*" OR hospital* OR clinic* OR "primary care" OR "health facilit*" OR "continuity of care" OR "service delivery" OR "access to care" OR demand OR hospitalization OR utilisation* OR utilization* OR coverage OR "maternal health services" OR "child health services" OR "health workforce" OR "occupational heat stress" |
| ProQuest (Environmental Sci & Public Policy) | flood* OR rain* OR heat* OR "high temperature*" OR "hot temperature*" OR overheating OR "temperature change*" OR "temperature rise" OR "hot weather" OR "extreme temperature*" OR "ambient temperature" OR "environmental temperature" OR exposure OR humidity OR "thermal discomfort" OR "nighttime temperatures"  AND  adapt* OR "risk reduction" OR DRR OR "hazard adaptation" OR "emergency preparedness" OR prepared* OR "evacuation strategy" OR "contingency planning" OR "nature-based solution*" OR "ecosystem-based adaptation" OR EbA OR "resilient infrastructure" OR "climate-proof*" OR "health system resilience" OR "early warning system*" OR "capacity building" OR "disaster response" OR "crisis management" OR strateg* OR framework*  AND  "health system*" OR "health service*" OR hospital* OR clinic* OR "primary care" OR "health workforce" OR "continuity of care" OR "maternal health services" OR "child health services" OR utilization OR coverage OR access OR delivery |

1. Correspondence: [Sisay.Debele@lshtm.ac.uk](mailto:Sisay.Debele@lshtm.ac.uk)
